# Analysis of Benzene Exposure in Gas Station Workers Using Trans,Trans-Muconic Acid

**DOI:** 10.3390/ijerph17155295

**Published:** 2020-07-23

**Authors:** Barbara Rodrigues Geraldino, Rafaella Ferreira Nascimento Nunes, Juliana Barroso Gomes, Isabela Giardini, Paula Vieira Baptista da Silva, Élida Campos, Katia Soares da Poça, Rocio Hassan, Ubirani Barros Otero, Marcia Sarpa

**Affiliations:** 1 Technical Area of Environment, Work and Cancer, Prevention and Surveillance Coordination, National Cancer Institute José Alencar Gomes da Silva–INCA, Rio de Janeiro CEP 20230-240, Brazil; rafaella.nunes@inca.gov.br (R.F.N.N.); bgomesjuliana@gmail.com (J.B.G.); giardini.isabela@gmail.com (I.G.); paulavbs.biomed@gmail.com (P.V.B.d.S.); elidacamp@gmail.com (É.C.); katia.soares@inca.gov.br (K.S.d.P.); uotero@inca.gov.br (U.B.O.); mmello@inca.gov.br (M.S.); 2 Environmental Mutagenesis Laboratory, Department of Biochemistry, Biomedical Institute, Federal University of Rio de Janeiro State (UNIRIO), Rio de Janeiro CEP 20211-040, Brazil; 3 Oncovirology Laboratory, Bone Marrow Transplantation Center (CEMO), National Cancer Institute José Alencar Gomes da Silva–INCA, Rio de Janeiro CEP 20230-130, Brazil; chassan@inca.gov.br

**Keywords:** benzene, gas station workers, t,t-MA

## Abstract

In Brazil, gas station workers are occupationally exposed to the benzene present in gasoline. Brazilian law indicates the use of trans,trans-muconic acid(t,t-MA) as a biomarker of benzene exposure. The aim of this study was to evaluate the level of exposure to benzene in gas station workers, through the quantification of t,t-MA present in urine. A total number of 269 gas station workers divided into 179 filling station attendants exposed by inhalation and dermal route and 90 convenience store workers exposed only by inhalation were included. A control group was formed by 100 office workers, without occupational exposure to benzene. The urinary levels of t,t-MA were evaluated by HPLC with a UV detector. Gas station workers showed higher mean values of t,t-MA (0.204 mg/g creatinine; 95% CI 0.170–0.237) than office workers (0.126 mg/g creatinine; 95% CI 0.0817–0.1693). T,t-MA levels were higher in convenience store workers exposed to gasoline only by inhalation (0.221 mg/g creatinine; 95% CI 0.160–0.282), than in those exposed to gasoline by inhalation and dermal route—filling station attendants (0.195 mg/g creatinine; 95% CI 0.155–0.235). Gas station workers with a higher level of t,t-MA had epistaxis. T,t-MA values were higher in the Downtown (0.15 mg/g creatinine) region’s workers than in the more affluent South Zone region’s workers (0.07 mg/g creatinine). Smoking habits influenced the urinary t,t-MA values, while the frequency of consumption of industrialized and frozen foods showed no influence.

## 1. Introduction

Benzene is an industrial chemical that demands huge concern from regulatory authorities around the world. According to the World Health Organization (WHO) and the Agency for Toxic Substances and Disease Registry (ATSDR), benzene is among the ten chemicals of greatest toxicological importance, and its exposure is considered to be a serious public health problem [1,2].

Benzene is used on a large scale in the world, and is a universal contaminant that is present in the atmospheres of rural and urban areas, and in higher concentrations in certain workplaces. Cigarette smoke and gasoline represent the two main sources of human exposure to benzene [3]. In Brazil, gasoline contains up to 1% benzene (concentration to volume) [4] in its composition.

Human exposure to benzene occurs orally, through dermal contact, or through the inhalation of benzene vapors present in the air [5]. The main form of absorption of benzene present in gasoline is through the respiratory tract, also being absorbed by the dermal route [6]. Depending on the degree of exposure and the frequency that the individual is subjected to, exposure to benzene causes a set of signs, symptoms and complications gathered in a chart called benzene poisoning, of which we can highlight local effects, such as mucosal irritations, edema pulmonary, hemorrhages, in addition to systemic effects such as dizziness, headache, nausea, tachycardia, tremors, convulsions, breathing difficulties, loss of consciousness and death [5]. Benzene also causes oxidative stress and oxidative damage associated with DNA; genotoxicity, inducing DNA damage and chromosomal changes; immunosuppression; and hematotoxicity [4], besides non-lymphocytic leukemia, including acute myeloid leukemia in adults. Positive associations have also been found with chronic lymphocytic leukemia, chronic myeloid leukemia, non-Hodgkin’s lymphoma, multiple myeloma and lung cancer [4], therefore, benzene was classified as a Group 1 carcinogen by the International Agency for Research on Cancer—IARC [7].

The myelotoxic and leukemogenic action of benzene has been attributed to their biotransformation products formed in bone marrow [8]. The biotransformation of benzene in the liver generates a variety of hydroxylated products. At the end of this process, benzene epoxide conjugated to glutathione will generate the urinary metabolite S-phenylmercapturic acid (S-PMA) [9,10,11] and the trans-trans-mucaldehyde, and after the action of the enzymes alcohol dehydrogenase and aldehyde dehydrogenase, will generate trans,trans-muconic acid (t,t-MA). It is estimated that a variation of 2.0 to 3.9% of the absorbed benzene is excreted as t,t-MA in the urine, and that the elimination half-life of this metabolite is 5.1 h [12].

T,t-MA and S-PMA have been used worldwide as biological exposure indicators (BEI) to benzene [8]. However, in Brazil, only the t,t-MA in the urine collected at the end of the working day is indicated as a biological indicator of exposure to benzene [13]. The use of t,t-MA as a biological indicator of benzene exposure has some advantages, such as the ease and analytical sensitivity of its urine determination [14]. Moreover, a linear correlation between the urinary t,t-MA and benzene environmental concentration has been described. Urinary t,t-MA values around 0.9 to 1.9 mg/g creatinine have been correlated with exposure to 1.0 ppm benzene [8]. The correlation between t,t-MA and benzene is detected at ambient levels below 1.0 ppm, the maximum concentration of benzene allowed by the Brazilian legislation [15].

Exposure to benzene, even at concentrations below 1 ppm, is potentially dangerous, because it is possible to observe changes in the blood counts of individuals chronically exposed to benzene at these concentrations [13]. In addition, due to its association with the development of cancer, the exposure of workers to benzene present in gasoline is worrying, since these workers are exposed to this agent around 8–9 h a day, 6–7 days a week, either by inhalation and/or the dermal route [6,16].

In Brazil, there are 40,544 gas stations, with 15,545 located in the Southeast Region. Of these, 2107 gas stations are present in the state of Rio de Janeiro, and have approximately 180,000 gas station workers [17]. Despite efforts to regulate benzene emissions, studies show that the concentration of atmospheric benzene in the Brazil gas stations is above the value of 5 pg m-3, established by Directive 2000/69 of the European Community (2000) [18,19]. It was observed that the concentrations of BTEX in the ambient air of gas stations in Rio de Janeiro (Brazil) were substantially higher than the average values in places with high vehicular flow, and these values were dependent on the number of fuel pumps at gas stations, and the number of filling operations and air circulation each day [19].

The exposure of gas station workers to benzene through ambient air can be substantially increased, due to the functions performed by them, such as: filling the vehicles, checking the fuel pump, receiving the fuel tanker truck, analyzing the control samples of the fuel received, unloading fuel from the tanker truck and measuring the fuel level in the underground tank of gas stations [20,21,22]. Gas station workers are among the second group of workers most potentially exposed to benzene in Brazil [23], and they are vulnerable to increased exposure to benzene present in the atmosphere of these locations.

Thus, the aim of the present study was to evaluate the level of exposure to the benzene of different groups of gas station workers using t,t-MA as exposure biomarker. The prevalence of signs and symptoms reported by workers was also assessed. Furthermore, it was verified whether the type of work performed by gas station attendants and whether the location of gas stations influenced workers’ trans,trans-muconic acid levels.

## 2. Materials and Methods

### 2.1. Socio-Geographical Characteristics

The present study was carried out in two distinct urban spaces, planning areas 1 and 2 (PA1 and PA2) of the Metropolitan region of Rio de Janeiro [24]. There are no differences in the types of work performed by workers at gas stations in regions PA1 and PA2. The difference that exists is related to the location of gas stations within the Rio de Janeiro city.

The central area (downtown) of Rio de Janeiro (PA1) is a highly urbanized, more commercial than residential area, with tall buildings, old houses, and population clusters, such as small and medium-sized favelas. In this region, the main roads that connect the city and the main express way are inserted, which result in intense vehicular traffic. Green areas occupy a limited space in this region.

On the other hand, the South Zone (PA2) is an area of great real estate valuation, housing most of the affluent population [25]. Besides its high infrastructure standards, this region has a milder microclimate, because it has a large variety of wooded areas, as well as natural landscapes, such as Rodrigo de Freitas Lagoon, Botanical Garden, and a coastal area with numerous beaches.

The marked differences at geographical characteristics of the PA1 and PA2 planning areas can interfere with air dispersion, potentially contributing to different levels of exposure the among the exposed works, leading to variable levels of t,t-MA urinary on the distinct urban spaces.

### 2.2. Study Design and Population

A cross-sectional epidemiological study was carried out with workers from 21 gas stations in the city of Rio de Janeiro, nine located in the Central (downtown) region, and 12 gas stations in the South Zone of the Rio de Janeiro city. In these gas stations, 319 workers were recruited and interviewed, and were divided into 2 groups: filling station attendants group, directly exposed to benzene, which supply vehicles and have direct inhalation and dermal contact with gasoline (filling station attendants, managers, lubricators and alike); and convenience store workers group, workers who did not work directly with the fuel, therefore are considered indirectly exposed to benzene in the workplace, only by inhalation of gasoline vapors (administrative workers, convenience store attendants and cleaning service). The activities developed by convenience store workers are serving customers, selling food and drinks, and cleaning or managing convenience stores.

The control group (office workers), that is, workers who were not occupationally exposed to benzene, comprised 116 individuals from administrative areas of the Brazilian National Cancer Institute (INCA) and the Federal University of the State of Rio de Janeiro (UNIRIO). The main characteristic that would make them eligible for the control group was not being occupationally exposed to benzene or gasoline.

For this particular study, 43 of 319 samples in the exposed group (gas station workers), and 16 of 116 samples in the control group (office workers) were excluded from the analysis because they were outside the acceptable limits creatinine range, between 0.3 g/L and 3.0 g/L [26]. Moreover, 7 individuals from the exposed group were excluded, because of an outlier t,t MA value. Therefore, a total of 100 office workers (control group) and 269 gas station workers were included in the analysis, 179 directly exposed by inhalation and dermal route (filling station attendants) and 90 exposed only by inhalation (convenience store workers).

In order to assess the signs and symptoms caused by exposure to benzene, only workers who worked at gas stations for more than 6 months were included in the study. Sampling of the stations was non-probabilistic, and in all establishments, employees aged >18 were invited to participate, regardless of their function. The study was approved by the INCA’s Ethics Committee (registry number 121/09), and volunteers were included in the research after signing their informed consent.

### 2.3. Collection of Data

After being recruited, participants responded to a questionnaire, in which socio-demographic and historical information about the worker’s life and exposure to chemical agents during the workday were collected. Due to the possible interference of some habits in the t,t-MA urinary levels, the influence of factors such as smoking, the consumption of industrialized foods containing sorbic acid (or any of its salts) and alcohol consumption were investigated.

The participants then underwent a clinical evaluation and were interviewed by a physician. The workers answered a clinical questionnaire, in which information was collected on the current, as well as the history of, complaints, signs and symptoms. In this study, the following variables were used (no/yes): asthenia, anxiety, convulsions, depression, dizziness, epistaxis, headache, insomnia, irritability or nervousness, somnolence, tremor, and weakness.

### 2.4. Sample Collection

In order to include a larger number of participants in the study, urine samples were collected at the end of the working day from all shifts of 8 h (departure at 6 am, 2 pm and 10 pm). Samples were collected once for each worker and the sample of each worker was analyzed separately. After collection, the urine samples were conditioned in a styrofoam box containing recyclable ice, kept cold, and transported to the laboratory. Samples from office workers (not occupationally exposed to benzene; control group), were collected at the end of the working day, and manipulated in a similar way to samples from the exposed group (gas station workers).

### 2.5. Evaluation of Urinary Creatinine

Measurements of urine creatinine levels were performed after 12 h of collection, by Jaffe’s reaction, as determined by the end-point colorimetric method (Bioclin^®^). All urine samples that presented creatinine values lower than 0.3 g/L or greater than 3.0 g/L were excluded from analysis, following the guidelines of the American Conference of Governmental Industrial Hygienists (ACGIH) [10,26].

### 2.6. Evaluation of Urinary t,t-MA

The urinary levels of the t,t-MA were evaluated by high efficiency liquid chromatography, a method initially proposed by Ducos et al. [27], modified by Paula [10] using a UV detector (HPLC-UV), equipped with a 5 μm Luna Phenomenex^®^ chromatography column (250 × 4.6 mm). The standard was obtained from Sigma-Aldrich^®^ and the normalization of results was adjusted by creatinine values. Samples were cleaned up by solid phase extraction (SPE), in Applied Separations^®^ cartridges, N+ Quaternary Amino (SAX), 500 mg/3 mL. SPE conditioning was as follows: 3 mL of methanol, 3 mL of ultrapure water. One ml of urine was added, followed by a pre-washing of 3 mL 1% acetic acid. Finally, elution occurred in 10% acetic acid (pH 2.7). Subsequently, an aliquot of 20 µL was injected into the HPLC by manual injection, and the chromatographic conditions are described below.

### 2.7. Chromatographic Conditions for t,t-MA Quantification

The mobile phase consisted of a 1% aqueous solution of acetic acid-methanol (90-10 *v/v*), pH 2.72 in a flow rate of 1.0 mL/min. The total time of the chromatographic run was 13 min; the detector was held at λ = 264 nm for reading and the column was maintained at 40 °C. The limit of detection was 0.0075 mg/L.

### 2.8. Statistical Analyses

A database was constructed with the information collected in the interviews and the results of the laboratory analyses. Descriptive analyses of some characteristics related to the study population were carried out. For continuous variables, the median, as a measure of central tendency for non-normal, asymmetric distribution was used. Other variables were presented as absolute values and percentages. Comparisons of the exposure groups were performed with the chi-square test. t,t-MA levels were analyzed as a continuous variable, with normality evaluated using the Kolmogorov–Smirnov test. In addition, t,t-MA values were categorized using reference values recommended by American Conference of Governmental Industrial Hygienists (ACGIH) (0.5 mg/g creatinine) [26]. Bivariate analyses were performed with the non-parametric Mann–Whitney test.

To determine the association between benzene exposure and urinary t,t-MA levels, a non-conditional logistic multivariate regression was performed. Variables associated with the benzene exposure and the t,t-MA levels in the bivariate analysis (*p*-values ≤ 0.20) were included in the models; *p*-values in the final model were considered significant at ≤0.05. However, given the relevance of sex, age, smoking, alcohol consumption and industrialized food, the final model was adjusted by these variables, regardless of statistical significance. Statistical analyzes were performed using the Statistical Package for Social Sciences (SPSS) for Windows—version 17.0 (SPSS Inc., Chicago, IL, USA).

## 3. Results

Table 1 shows the characteristics of the study population. The analysis between groups was performed by comparing office workers and gas station workers, which were divided between convenience store workers and filling station attendants. Groups were homogeneous in respect of alcohol (*p* = 0.30) and processed food (*p* = 0.22) consumption. However, some differences were observed between groups that are worth mentioning. In general, filling station attendants smoked more (19.0%) compared to office workers (6.0%) and convenience store workers (7.8%). The average age of filling station attendants (37 years old) was similar to office workers (39 years old), but different to those in the convenience store (30 years old). Filling station attendants were mostly male (88.8%), different to convenience store workers, who were mostly women (70.0%). Differences between groups for these variables were statistically significant (*p* < 0.05).

Urinary t,t-MA levels (mg/g creatinine) of the study population according to exposure category, smoking, alcohol consumption and processed foods are shown in Table 2. An analysis of the 95th percentile values show that 95% of the urine samples from the control group (office workers) had values of urinary t,t-MA lower than 0.5 mg/g creatinine (Table 2), which represents the level of biomarker that is most likely to be observed in urine collected from healthy workers who have been exposed by inhalation to benzene at environmental concentrations (limit value adopted by ACGIH of 0.5 ppm) [26]. The values of t,t-MA (95th percentile) for the group of gas station workers (0.785 mg/g creatinine) doubled the values found in the office workers (0.449 mg/g creatinine) control group.

A comparison of the median levels of urinary t,t-MA (mg/g creatinine) in gas station workers and office workers (control group) disclosed higher median urinary t,t-MA values in occupational exposed individuals (0.10 vs. 0.05 mg/g creatinine; *p* < 0.01, Mann–Whitney test). Higher levels were also observed in smokers compared with non-smokers (0.150 vs. 0.075 mg/g creatinine, respectively; *p* = 0.004). No differences were observed with respect to alcohol or industrialized food consumption (Table 2).

Levels of urinary t,t-MA were higher in the convenience store workers (0.221 mg/g creatinine; 95% CI 0.160–0.282) than in the filling station attendants (0.195 mg/g creatinine; 95% CI 0.155–0.235), although the difference did not reach statistical significance (*p* = 0.463).

In order to model the effect of exposure on t,t-MA levels, a logistic regression, adjusted for sex, age, and tobacco, alcohol and processed food consumption was performed (Table 3), showing seven times higher odds of elevated urinary t,t-MA levels in the gas station workers group compared to the office workers group (control group).

The presence of signs and symptoms were assessed in workers occupationally exposed to benzene, and compared to urinary t,t-MA levels (Table 4). The frequency of epistaxis was higher in workers with high t,t-MA levels (0.170; 0.05–1.59), than in workers with a lower quantification of urinary t,t-MA (0.090; 0.00–1.40), with significant differences between groups (*p* = 0.045, Mann–Whitney test). With regard to asthenia, though it also shows a statistically significant difference between groups (*p* = 0.047, Mann–Whitney test), it was more frequent among workers with lower levels of t,t-MA (0.060; 0.00–0.93 against 0.100; 0.00–1.59). Convulsions, depression, dizziness, headache, insomnia, irritability or nervousness, somnolence, tremor and weakness were also evaluated, but the results did not disclosed statistically significant differences in relation to urinary t,t-MA levels, as can be seen in Table 4.

When the geographic location of the gas stations was taken into account, workers at the gas stations in the South Zone of the Rio de Janeiro city presented median t,t-MA values about 50% lower than those workers at gas stations located in the Downtown area ( *p* < 0.01; Mann–Whitney test) (Table 5).

## 4. Discussion

In this work, we evaluated the level of exposure to benzene of different groups of workers at gas stations in Rio de Janeiro city, using urinary t,t-MA as exposure biomarkers, and found that the urine samples from gas station workers occupationally exposed to benzene exhibited t,t-MA levels significantly higher than the control group, formed by office workers. Moreover, the geographic location of the gas stations impacted the risk of exposure, with workers at the gas stations located in the more affluent and ecologically conserved South Zone region, presenting median t,t-MA values about 50% lower than those at stations located in the City Center (Downtown).

It is important to have in mind that benzene is a carcinogenic substance [7], therefore, there is no safe exposure limit, and limit values should not be established for biological exposure indicators (BEI), such as t,t-MA. However, ACGIH (2012) [26] and the Ordinance 34 (2001) [13] of the Ministry of Labor and Employment of Brazil suggested that, in workers occupationally exposed to benzene, the concentration of t,t-MA should be below 0.5 mg t,t-MA/g creatinine. The presence of urinary t,t-MA (even below 0.5 mg/g creatinine) in non-occupationally exposed people is generally attributed to the extensive environmental pollution by benzene that arises from sources such as smoking and urban pollution [28]. In the present study, although the levels of urinary t,t-MA found in gas station workers (occupationally exposed to benzene) were lower than the “biological exposure indicators” suggested by ACGIH and Ordinance 34, the chance of having altered levels of urinary t,t-MA is 7 times greater in the occupationally exposed group compared to the control group, even when adjusted by sex, age, smoking, alcohol consumption and industrialized food. Despite all the limitations of t-t-AM as a biomarker of exposure to benzene, this result strongly suggests that increases in its levels are significantly associated with occupational exposure to benzene.

Gas station workers, in general, are exposed to different solvents present in the various types of fuels marketed, in addition to environmental pollution, since the work occurs in open environments [29]. Filling station attendants, one of the main work forces at these facilities, perform activities such as vehicle filling, checking fuel levels in vehicle storage compartments, and changing vehicle fluids, such as oils, lubricators. During these tasks, even in an unintentional way, there is an emission of the vapors present in the fuels, mainly compounds of high volatility, as is the case of benzene. Thus, not only employees participating in the supply of vehicles, but also other employees listed in other occupations, end up suffering exposure to the solvents present in the fuels by indirect exposure, that is, by inhalation. In the workers exposed only by inhalation (convenience store workers) values of t,t-MA (mean = 0.221 mg/g creatinine) were higher than filling station attendants (mean = 0.195 mg/g creatinine), although without statistical significance, suggesting that the indirectly exposed convenience store workers are as exposed as gas station workers, who work on the runway of fuel resale points and are exposed by inhalation and the dermal route.

Gas stations share space with other types of commerce establishments, such as convenience stores and snack bars. Ali et al. (2013) [30] have shown that mechanical ventilation systems are not efficient in dispersing volatile organic pollutants. Thus, in those establishments, it is possible to find a concentration of atmospheric pollutants indoors, at higher levels than in open environments [31]. In addition, convenience store workers, exposed only by inhalation, remain inside convenience stores all day, which use air conditioning systems without renewal or internal air exchange. This is particularly worrisome, since these compounds have high vapor pressure, being easily vaporized in the condition of ambient temperature and pressure.

Our results suggest that the gas station environments are potentially hazardous in terms of exposure to benzene. Although the exposure time is small for customers who supply on a weekly basis and use the surrounding trade, the frequency of exposure causes the risk to be much higher for workers at these locations. Morales Terrés et al. (2010) [32] evaluated atmospheric contamination around gas stations in the southeastern Iberian Peninsula, and concluded that, the greater the distance from the gas station, the lower the rate of pollutant concentration, including benzene, indicating that the environment near the gas stations fuel is more contaminated.

A study by Parvin (2014) [33] found that the levels of benzene in convenience stores were always higher than that established by legislation in places where smoking was allowed. However, even in environments where cigarette use was banned, benzene levels exceeded the reference value. In our study, higher levels of t,t-MA was observed in smokers (0.15 mg/g creatinine) when compared with non-smokers (0.08 mg/g creatinine). This result is in line with previously published results, showing that smokers occupationally exposed to benzene excrete more t,t-MA in the urine than non-smokers [33,34,35].

Moreover, it has been reported that the consumption of alcoholic beverages does not seem to have significantly influenced urinary t,t-MA values [10]. We have also found no association between levels of urinary t,t-MA and the alcoholic beverages. Similarly, since literature reports that diet rich in sorbic acid can generate metabolic compounds similar to t,t-MA [36], we decided to adjust the multivariate model with the industrialized food consumption variable, but the consumption of industrialized foods did not influence the urinary t,t-MA values.

In the present study, the levels of urinary t,t-MA found in the occupationally exposed to benzene group (gas station workers; 0.204 mg/g creatinine) and non-occupationally exposed to benzene group (office workers; 0.126 mg/g creatinine) were similar to other studies available in the literature with gas station workers, as well as other sectors.

Sauer et al. (2018) [37] assessed the levels of t,t-MA in gas station workers and office workers (control group). The authors observed that the median of t,t-MA was about three higher in the exposed group (P50 = 0.243 mg/g creatinine) when compared to the group not occupationally exposed to benzene (P50 = 0.074 mg/g creatinine). In the present study, the median urinary t,t-MA values observed in the exposed group (0.100 mg/g creatinine) was twice as high as the unexposed group (0.050 mg/g creatinine). Although the median urinary t,t-MA of the group occupationally exposed to benzene in both studies was lower than the biological exposure indicator (BEI) suggested by ACGIH (0.5 mg/g creatinine) (ACGIH, 2012) [26], in both studies, a percentage of gas station workers with values higher than the BEI (14.07% versus 33.78% in the study by Sauer et al.) was reported. It is worth mentioning that the sample number, geographical and seasonal differences can account for the differences found between the studies.

On the other hand, a study carried out in the metropolitan region of Belo Horizonte (MG/Brazil), that evaluated 53 individuals, with 31 workers exposed to benzene (technicians from the fuel testing laboratory) and 22 individuals not occupationally exposed to benzene (administrative agents), showed that the urinary t,t-MA (mg/g creatinine) was significantly higher in the exposed group (1.13 ± 0.45), when compared with the not occupationally exposed group (0.44 ± 0.33) [38]. These values were higher than those found in the present study, which may be related to different working processes of the exposed group (laboratory technician versus gas stations attendants) and the sample number.

Signs and symptoms related to benzene poisoning were assessed, and workers who had higher levels of urinary t,t-MA reported more frequent nasal bleeding (epistaxis) than workers with lower levels of urinary t,t-MA. Our results showed that urinary t,t-MA values were about twice as high in individuals who claimed to manifest epistaxis (0.170 mg/g creatinine), when compared to those who did not present this symptom (0.090 mg/g creatinine). This result is consistent with that found by Rianto et al. (2018) [39], who observed a positive correlation between the working time of gas station workers and the dysfunction in mucociliary transport ascribed to the continuous inhalation of solvents, including benzene. This indicates that the main route of exposure to this organic solvent present in gasoline generates health damage in continuously and permanently exposed workers. Besides that, as chronic exposure to low concentrations of benzene can contribute to bone marrow damage, it is plausible that only those workers with the highest concentrations of urinary t,t-MA have a higher frequency of epistaxis. Although environmental exposure to benzene at gas stations is considered low, the concentration of benzene in the air may be enough to allow absorption, causing changes in hematopoiesis in the long term. Therefore, nasal bleeding can occur as the bone marrow damage develops, and can be seen precisely in those with the highest levels of the urinary metabolite [7,40].

Bleeding from the nose (epistaxis), gums or mucous membranes and petechiae have been reported to occur as the exposure condition progresses [40], which was also found in the present study for epistaxis. Yu et al. (2004) [41], in a study of workers in printing factories, observed that individuals exposed to organic solvents have a drunken feeling (OR 2.9; 95% CI 1.2–7.0), abnormal smell (OR 1.6; 95% CI 1.0–2.6), reduced sense of smell (OR 2.4; 95% CI 1.1–4.9), complaint about the eyes (OR 1.6; 95% CI 1.1–2.3) or nose irritation symptoms (OR 1.5; 95% CI 1.0–2.1), when compared with those not using solvents in their jobs.

Asthenia is a known symptom of benzene poisoning [14], although in the present study, it was more frequent among workers with the lowest levels of urinary t,t-MA. Data from the literature indicate that the first signs and symptoms related to benzene exposure are varied, often not easily perceived as specific. The first subjective complaints are usually headache, dizziness and loss of appetite, but increased pulse rate, blood pressure drops, shortness of breath, and weakness may also occur.

Another result worth mentioning is that the workers at the stations located in the more affluent part of the city (South zone) had significantly lower median t,t-MA values than the workers at stations located in the more urbanized and poor region (Downtown region) (median = 0.07; median = 0.15; *p*-value = 0.002, respectively). Since the concentrations of volatile organic compounds (VOCs) in the environment are affected by fuel use, vehicle type and age, traffic flow and speeds, as well as the city’s environmental conditions [33], it is likely that the environmental differences between the gas stations directly influenced the increase of the fuel vapors, and as a consequence, the worker’s exposure. Chaiklieng et al. (2019) [42] evaluated the level of benzene in the air in 150 gas station workers in three different areas (urban, sub-urban and rural), and observed a higher concentration of benzene in the air in sub-urban area than other areas, presenting a higher risk of cancer. The study by Milazzo et al. (2019) [43] evaluated the influence of more wooded areas in relation to the concentration of BTEX in the air in parks, and in homes close to parks in urban and rural areas in the United States. They observed lower concentrations of BTEX in parks than in residential areas, suggesting that areas with more vegetation may help to dissipate the amount of BTEX, by helping to reduce exposure to the compounds.

It is known that t,t-MA is a sensitive biological exposure indicators, but exhibits medium specificity, and its concentration is influenced by smoking, simultaneous exposure to toluene, or the intake of sorbic acid and its salts in the diet [27,44,45,46]. In this study, the levels of urinary t,t-MA have shown to be useful to monitor benzene occupational exposure in gas station workers, since a control group not occupationally exposed to benzene was included in the study. In fact, the comparison of the results found in the gas station workers only with the reference value (0.5 mg/g creatinine) indicated by ACGIH [26] and Ordinance 34 (Brazil) [13] was not able to clearly disclose the degree of exposure of these workers.

Furthermore, it is important to acknowledge that one of the limitations of this study is related to discrepancies regarding some general characteristics between the gas station workers group (occupationally exposed group) and office workers (control group). Desirably, the control group (not occupationally exposed to benzene) should be chosen by criteria of correspondence to the exposed group (age, sex, geographic space, income, schooling). Nonetheless, we find an enormous difficulty in finding a group where the main variable was only benzene exposure. Therefore, to reduce the observed differences, careful adjustment for sex, age, smoking, consumption of alcoholic beverages and industrialized food consumption was made in multivariate models.

## 5. Conclusions

This study shows that the levels of urinary t,t-MA were lower in the not occupationally exposed group than in gas station workers, and the frequency of epistaxis increased in the last one. In addition, gas station workers in Rio de Janeiro downtown had higher t,t-MA values than in the more affluent South Zone. The results found in this study allow one to understand the levels of exposure to benzene to which these workers are susceptible daily, and can be used to support changes in current legislation, assist decision making, and modify the reality of work, in order to increase the protection of workers’ health.

## Figures and Tables

**Table 1:** Socio-demographic characteristics of office workers (control group) and gas station workers exposed to benzene in Rio de Janeiro (Brazil), 2015–2017.

Variables	Office Workers * N = 100 (%)	Gas Station Workers **		*p*-Value
		Convenience Store Workers N = 90 (%)	Filling Station Attendants N = 179 (%)	
**Age, years-median, (min–max)**	39 (20–61)	30 (20–67)	37 (20–70)	0.000
**Sex**				
Men	46 (46.0)	27 (30.0)	159 (88.8)	<0.001
Woman	54 (54.0)	63 (70.0)	20 (11.2)	
**Smoking**				
Non-smoker	86 (86.0)	69 (76.7)	114 (63.7)	0.001
Ex-smoker	8 (8.0)	14 (15.6)	31 (17.3)	
Smoker	6 (6.0)	7 (7.8)	34 (19.0)	
**Alcohol consumption**				
No	34 (34.0)	38 (42.2)	58 (32.4)	0.302
Yes	66 (66.0)	52 (57.8)	121 (67.6)	
**Industrialized foods consumption**				
No	8 (8.1)	4 (4.5)	11 (6.2)	0.218
1–2 times a week	20 (20.2)	28 (31.5)	58 (33.0)	
>2 times a week	62 (62.6)	54 (60.7)	98 (55.7)	
Rarely	9 (9.1)	3 (3.4)	9 (5.1)	

*Notes*: Chi-square test used for comparison between groups in categorical variables. * Not occupationally exposed to benzene. ** Occupationally exposed to benzene.

**Table 2:** Distribution and comparison of urinary t,t-MA (mg/g creatinine) levels in office workers (control group) and gas station workers exposed to benzene in Rio de Janeiro (Brazil), 2015–2017.

	N	Mean	SD	MIN	MAX	P25	P50	P75	P95	*p*-Value ^#^
Office workers *	100	0.126	0.221	<LOD	1.630	0.020	0.050	0.138	0.449	0.012
Gas station workers **	269	0.204	0.277	<LOD	1.590	0.040	0.100	0.255	0.785	
Filling station attendants	179	0.195	0.270	<LOD	1.400	0.040	0.090	0.250	0.860	0.463
Convenience store workers	90	0.221	0.293	<LOD	1.590	0.038	0.105	0.283	0.785	
**Tobacco consumption**										
No	322	0.167	0.249	<LOD	1.630	0.030	0.075	0.210	0.580	0.004
Yes	47	0.286	0.342	<LOD	1.400	0.050	0.150	0.370	1.220	
**Cigarettes consumed per day**										
<10	24	0.208	0.273	<LOD	1.190	0.030	0.075	0.298	1.035	0.115
>10	23	0.366	0.392	<LOD	1.400	0.110	0.240	0.560	1.368	
**Alcohol consumption**										
No	130	0.183	0.264	<LOD	1.630	0.030	0.080	0.225	0.668	0.990
Yes	239	0.182	0.267	<LOD	1.590	0.030	0.080	0.220	0.680	
**Industrialized food**										
No	23	0.206	0.262	<LOD	1.090	0.040	0.070	0.340	1.008	0.190
1–2 times a week	106	0.158	0.210	<LOD	1.090	0.020	0.060	0.240	0.573	
>2 times a week	214	0.199	0.297	<LOD	1.630	0.030	0.090	0.220	0.993	
Rarely	21	0.084	0.079	<LOD	0.029	0.0150	0.060	0.120	0.285	

t,t-MA: trans,trans-muconic acid. Mean: arithmetic mean; SD: standard deviation; MIN: minimum; MAX: maximum; LOD: below the detection limit. P: percentile. ^#^ Mann–Whitney test. * Not occupationally exposed to benzene; ** Occupationally exposed to benzene.

**Table 3:** Logistic regression analysis of occupational exposure to benzene and urinary levels of t,t-MA (mg/g creatinine) in office workers (control group) and gas station workers exposed to benzene in Rio de Janeiro (Brazil), 2015–2017.

Category of Exposure	Logistic Regression					
	Crude OR	*p*-Value	95% CI	Adjusted OR	95% CI	*p*-Value
Office workers *	1.0	0.005		1.0		0.010
Gas station workers **	8.03		1.89–33.92	6.98	1.60–30.36	

CI 95%: Confidence interval 95%; OR: odds ratio. Adjusted OR: sex, age, and tobacco, alcohol, and industrialized food consumption. *p*-values crude and adjusted OR ≤ 0.01. * Not occupationally exposed to benzene; ** Occupationally exposed to benzene.

**Table 4:** Correlation between the signs and symptoms of benzene poisoning and the levels of urinary t,t-MA in gas station workers occupationally exposed to benzene through gasoline in Rio de Janeiro (Brazil), 2015–2017.

		N	t,t-MA (Median/Min;Max)	*p*-Value ^#^
**Anxiety**				
	No	135	0.110 (0.00; 1.59)	0.053
	Yes	127	0.075 (0.00; 1.40)	
**Asthenia**				
	No	219	0.100 (0.00; 21.59)	0.047
	Yes	43	0.060 (0.00; 0.93)	
**Convulsions**				
	No	259	0.100 (0.00; 1.59)	0.455
	Yes	3	0.060 (0.04; 0.09)	
**Depression**				
	No	183	0.100 (0.00; 1.59)	0.574
	Yes	79	0.080 (0.00; 1.40)	
**Dizziness**				
	No	189	0.100 (0.00; 1.40)	0.277
	Yes	73	0.080 (0.00; 1.59)	
**Epistaxis**				
	No	246	0.090 (0.00; 1.40)	0.045
	Yes	16	0.170 (0.05; 1.59)	
**Headache**				
	No	159	0.100 (0.00; 1.40)	0.286
	Yes	103	0.080 (0.00; 1.59)	
**Insomnia**				
	No	194	0.090 (0.00; 1.59)	0.442
	Yes	68	0.130 (0.00; 1.40)	
**Irritability/nervousness**				
	No	167	0.100 (0.00; 1.59)	
	Yes	95	0.100 (0.00; 1.19)	0.903
**Somnolence**				
	No	148	0.095 (0.00; 1.34)	0.845
	Yes	114	0.100 (0.00; 1.59)	
**Tremor**				
	No	230	0.100 (0.00; 1.59)	0.950
	Yes	32	0.075 (0.00; 1.40)	
**Weakness**				
	No	206	0.100 (0.00; 1.59)	0.279
	Yes	56	0.080 (0.00; 1.18)	

^#^ Mann–Whitney test.

**Table 5:** Levels of urinary t,t-MA in gas station workers exposed to gasoline at South Zone and City Center (Downtown) in Rio de Janeiro (Brazil), 2015–2017.

Gas Station Workers		South Zone	Center (Downtown)	
		N = 173 (%)	N = 97 (%)	
		**Median**	**Median**	***p*-Value *^§^***
t,t-MA (mg/g de creatinine)		0.07	0.15	0.002

t,t-MA: trans,trans-muconic acid; ^§^ Mann–Whitney test.
